# Circadian Proteins CLOCK and BMAL1 in the Chromatoid Body, a RNA Processing Granule of Male Germ Cells

**DOI:** 10.1371/journal.pone.0042695

**Published:** 2012-08-10

**Authors:** Rita L. Peruquetti, Sara de Mateo, Paolo Sassone-Corsi

**Affiliations:** Center for Epigenetics and Metabolism, School of Medicine, University of California Irvine, Irvine, California, United States of America; Institute of Genetics and Molecular and Cellular Biology, France

## Abstract

Spermatogenesis is a complex differentiation process that involves genetic and epigenetic regulation, sophisticated hormonal control, and extensive structural changes in male germ cells. RNA nuclear and cytoplasmic bodies appear to be critical for the progress of spermatogenesis. The chromatoid body (CB) is a cytoplasmic organelle playing an important role in RNA post-transcriptional and translation regulation during the late steps of germ cell differentiation. The CB is also important for fertility determination since mutations of genes encoding its components cause infertility by spermatogenesis arrest. Targeted ablation of the *Bmal1* and *Clock* genes, which encode central regulators of the circadian clock also result in fertility defects caused by problems other than spermatogenesis alterations. We show that the circadian proteins CLOCK and BMAL1 are localized in the CB in a stage-specific manner of germ cells. Both BMAL1 and CLOCK proteins physically interact with the ATP-dependent DEAD-box RNA helicase MVH (mouse VASA homolog), a hallmark component of the CB. BMAL1 is differentially expressed during the spermatogenic cycle of seminiferous tubules, and *Bmal1* and *Clock* deficient mice display significant CB morphological alterations due to BMAL1 ablation or low expression. These findings suggest that both BMAL1 and CLOCK contribute to CB assembly and physiology, raising questions on the role of the circadian clock in reproduction and on the molecular function that CLOCK and BMAL1 could potentially have in the CB assembly and physiology.

## Introduction

Spermatogenesis, the differentiation program of male germ cells, is characterized by a number of unique and remarkable features. This process involves cellular proliferation over repeated mitotic divisions, duplication of chromosomes, genetic recombination through crossing-over, reduction-division by meiosis to produce haploid spermatids, and terminal differentiation of the spermatids into spermatozoon, through a process called spermiogenesis [1]. The spermiogenesis process consists of intensive condensation of the chromatin (due to replacement of histone proteins by protamines), a substantial reduction in the cytoplasm volume (caused by extrusion of a large cytoplasmic vesicle - the residual body), and formation of a tail [2]. Development of male germ cells is also controlled by a distinct regulatory program involving sophisticated hormonal control from the hypothalamic-pituitary axis [3] and by a highly-specialized gene expression program, which involves complex epigenetic reprogramming at the level of histone modification and DNA methylation [4]–[6]. Furthermore, nuclear and cytoplasmic bodies typical of germ cells seem to play important roles during the gametogenesis process [7]. One of the most intriguing cytoplasmic structures of male germ cells is the so called chromatoid body (CB), which seems to be active from the beginning of the meiotic division, playing a role in the promotion of chromosomal synapsis and XY body formation [8] to the last steps of male germ cell differentiation, when round haploid spermatids differentiate into mature spermatozoon. The CB is a cloud-like cytoplasmic granule visible in pachytene spermatocytes and spermatids. Recent studies focused on the molecular composition and functions of the CB suggest that this structure acts as a subcellular center of different RNA-processing pathways, and centralizes post-transcriptional mRNA control in the cytoplasm of haploid male germ cells [9]–[11]. We have proposed that the CB shares important similarities with the processing bodies (P-bodies) of somatic cells [11], although while there are multiple P-bodies in somatic cells, round spermatids contain a single CB [10]. The central role of the CB in spermatogenesis is demonstrated by targeted gene ablation of its components in the mouse. Indeed, mutation of the genes encoding the ATP-dependent DEAD-box RNA helicase VASA (or MVH – mouse VASA homologue), and the mouse Argonaute/PIWI family RNA-binding proteins (MIWI), cause male sterility by spermatogenesis arrest [12], [13].

Environmental, physiological and molecular factors governed by seasonal and circadian rhythms have been associated to reproduction efficacy [14]. Most organisms restrict their activity to the night or day, making them nocturnal or diurnal, respectively [15]. At the molecular level, the circadian clock consists of transcriptional and post-translational feedback loops generated by a set of interplaying regulatory proteins. CLOCK and BMAL1 are core clock components that function as transcription factors by heterodimerizing through the PAS domain and inducing the expression of clock-controlled genes that present E-boxes in their promoters [16]. Circadian rhythms impact fertility by integrating daily and seasonal environmental signals to coordinate various processes involved in ovulation, sexual hormone production and secretion, mating, embryo development and parturition [17].

Studies have explored the possible role of clock proteins in reproduction [18], [19], including those showing that *Bmal1* and *Clock*-null male mice are sterile caused by deficiencies in steroidogenesis and exhibit a mildly reduction on fertility caused by problems not directly related to the spermatogenesis process, respectively [20], [19]. Here we have investigated the CLOCK and BMAL1 proteins subcolocalization in post-meiotic male germ cells and found that they accumulate in the CB, interacting with MVH, an essential element of the CB implicated in time-dependent RNA binding and control [12], [21]. The expression of CLOCK and BMAL1 temporally overlaps with CB proteins in round spermatids from seminiferous tubules at stages IV-VI of the spermatogenic cycle. Strikingly, ablation of BMAL1 generates significant CB morphological alterations. Our findings reveal the presence of clock proteins within a RNA processing granule in male germ cells, suggesting their possible implication in RNA metabolism during male gametogenesis.

## Materials and Methods

### Animal and Tissue Collection

Generation of *Bmal1*–deficient (*Bmal1* KO) mice [22] and *Clock*–deficient (*Clock* KO) mice [23] has been previously described. All comparative analyses using KO mice included their respective wild type (WT) littermates. Mice were maintained on a L12:D12 (12 h light–12 h dark) cycle and sacrificed using the CO_2_ inhalation method followed by cervical dislocation. Testes were collected and prepared for experimental procedures.

### Ethics Statement

The UCI Animal Welfare assurance number is A3416-01. All research involving vertebrate animals has been performed under protocols approved by the Institutional Animal Care and Use Committee (IACUC) (IACUC protocol number: 2006/2699). Animals were monitored on a daily basis for signs of distress, pain, and/or infection, and were given *ad libitum* access to food and water. Cages were cleaned on a weekly basis and/or when visibly soiled to maintain a clean environment. All husbandry procedures and welfare policies were conducted according to the Guide for the Care and Use of Laboratory Animals, set forth by the Institute of Laboratory Animal Resources, Commission on Life Sciences, and National Research Council.

**Figure 1:**
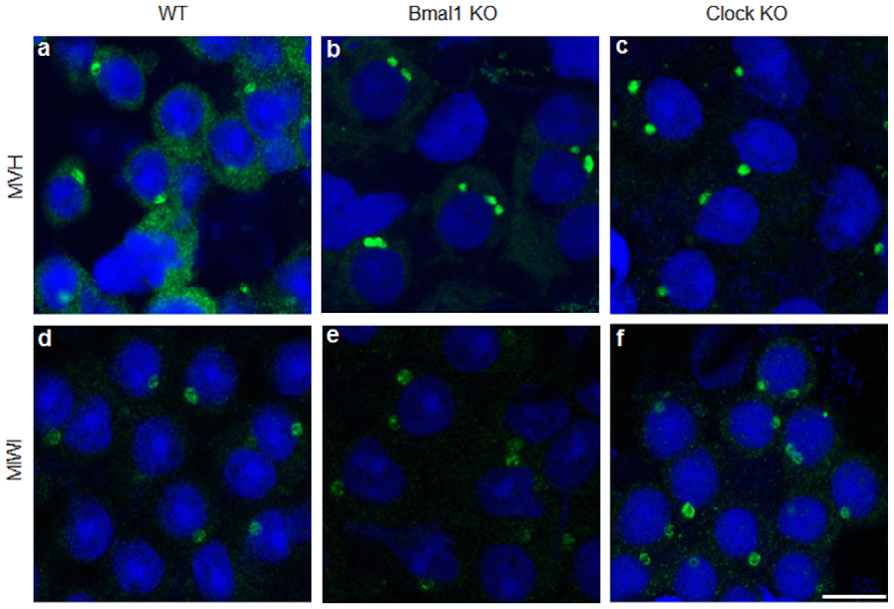
Alteration in the number of CB spots in *Bmal1* KO and *Clock* KO round spermatids. (a) and (d): Wild type (WT) round spermatids. (b) and (e): *Bmal1* knockout (KO) round spermatids. (c) and (f): *Clock* knockout (KO) round spermatids. Squash preparations of stages IV–VI seminiferous tubules were stained with anti-MVH antibody (a–c) (Green) and with anti-MIWI antibody (d–f) (Green). Two or three CB spots were observed in the cytoplasm of *Bmal1* KO and *Clock* KO spermatids but not in the cytoplasm of WT spermatids. DNA was counter-stained by DRAQ5 (Blue). Scale bar: 10 µm.

**Figure 2:**
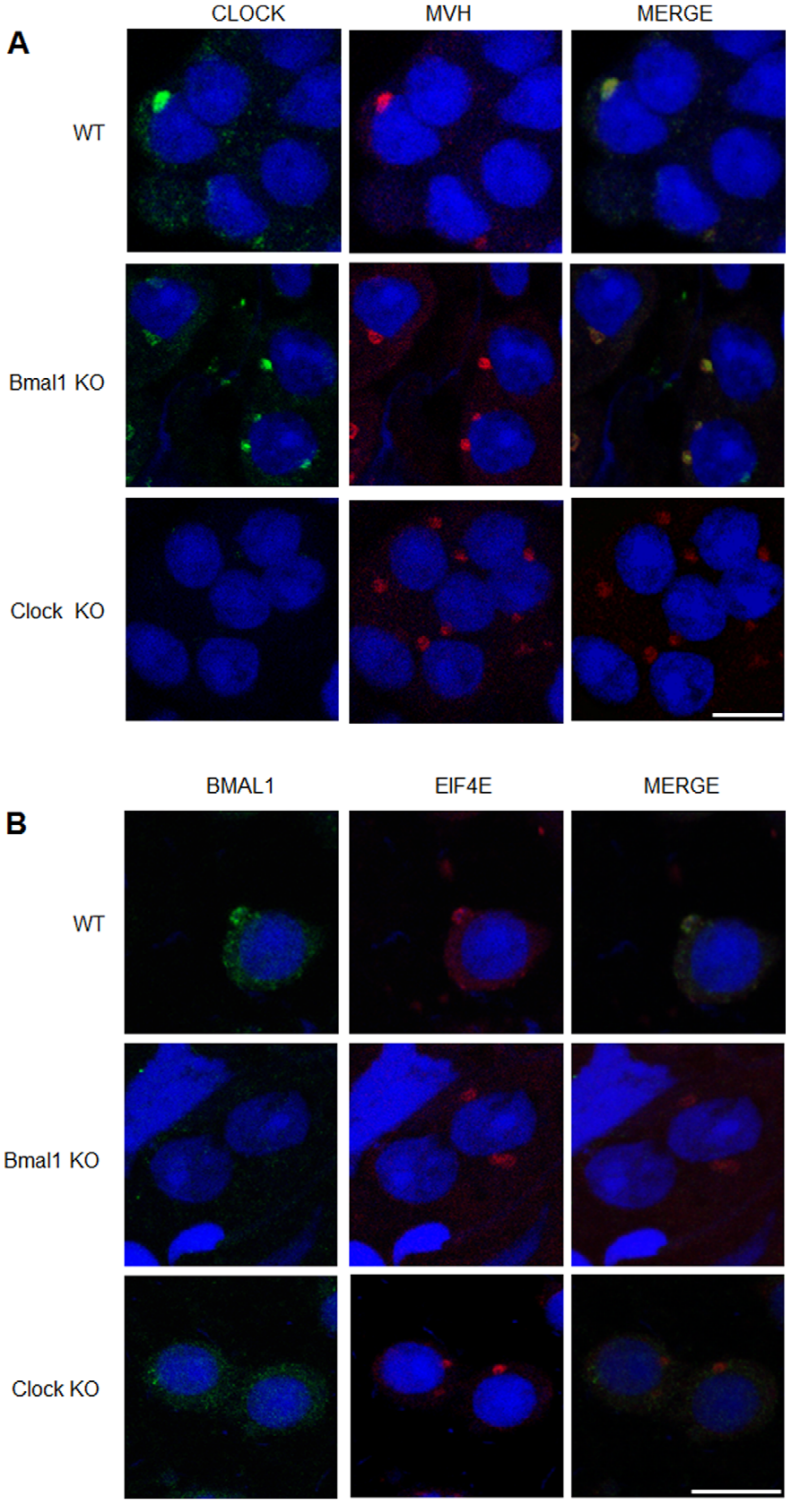
CLOCK and BMAL1 co-localize with CB proteins. (A) Squash preparations of stages IV-VI WT, *Bmal1* KO and *Clock* KO seminiferous tubules were stained with anti-CLOCK antibody (Green) and anti-MVH antibody (Red). The co-localization between CLOCK and MVH was observed in the WT and *Bmal1* KO round spermatids. (B) Squash preparations of stages IV-VI WT, *Bmal1* KO and *Clock* KO seminiferous tubules were stained with anti-BMAL1 antibody (Green) and anti-EIF4e antibody (Red). The co-localization between BMAL1 and EIF4e was observed only in the WT round spermatids. DNA was counter-stained by DRAQ5 (Blue). Scale bar: 10 µm.

**Figure 3:**
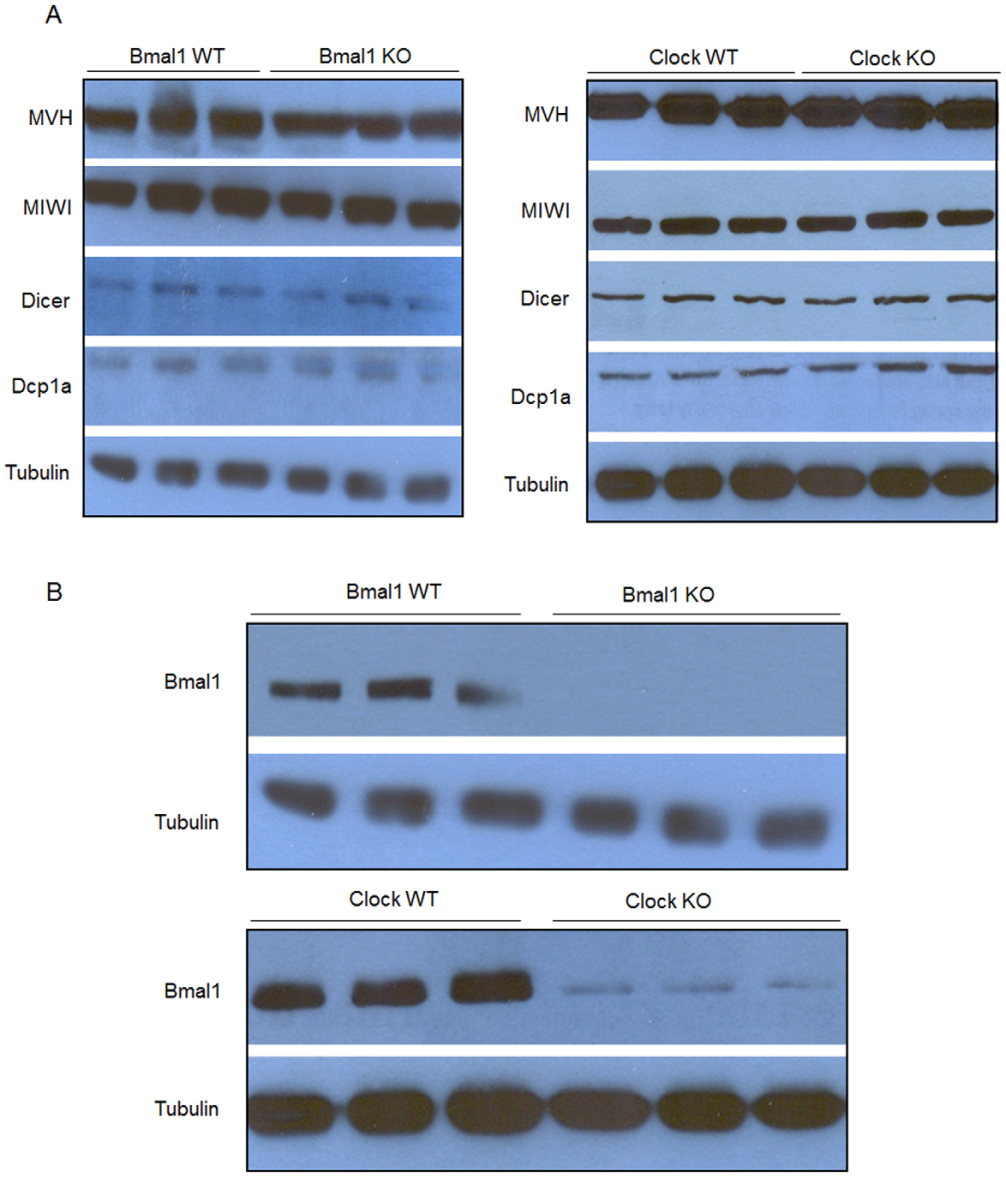
Total proteins were extracted from testis of WT, *Bmal1* KO and *Clock* KO mice. Electrophoretically separated denatured proteins were transferred to nitrocellulose membranes and (A) immunoblotted with antibodies against CB markers: anti-MVH, anti-MIWI, anti-DCP1a, and anti-DICER. No differences in the levels of CB proteins of WT mice and theirs respective knockouts; (B) immunobloted with antibody anti-BMAL1. *Clock* KO mice showed a decrease in BMAL1 expression in the testis. Tubulin served as a loading control.

### Immunofluorescence

Preparation of stage-specific squash slides was previously described [24]. For immunofluorescence, the squash preparations were postfixed with 4%PFA and then permeabilized with 0.2% Triton X-100 for 5 minutes. Non-specific sites were blocked by incubating slides in 5% BSA for 60 minutes. The primary antibody incubation was carried out at 4°C in 5% BSA solution with anti-MVH rabbit polyclonal antibody (1∶200), anti-MIWI rabbit polyclonal antibody (Cell Signaling – G82) (1∶200), anti-CLOCK goat polyclonal antibody (Santa Cruz Biotechnology, Inc. – S-19) (1∶200), anti-BMAL1 rabbit polyclonal antibody (Abcam – ab93806) (1∶200), and anti-EIF4E mouse monoclonal antibody (Abgent – AM1852a) (1∶200). Alexa Fluor 488 goat anti-rabbit IgG, Alexa Fluor 488 donkey anti-goat IgG, Alexa Fluor 546 goat anti-mouse IgG and Alexa Fluor 546 goat anti-rabbit IgG were used as secondary antibodies. Nuclei were stained using DRAQ5 solution (Biostatus Limited) and preparations were mounted in Vectashield (Vector Laboratories). Slides containing squash preparations were analyzed by confocal microscopy TCS SP5, and data were collected using Leica Application Suíte – Advanced Fluorescense (LAF AF) 1.8.2 Build 1465 (1997–2007).

**Figure 4:**
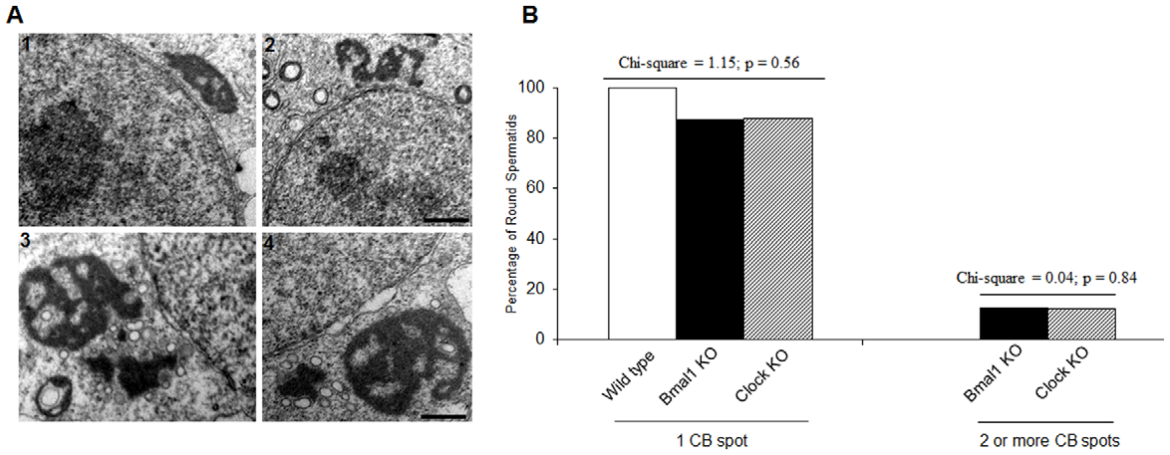
Alteration in the number of CB spots in the round spermatids from *Bmal1* KO and *Clock* KO mice. (A) Electron microscopy was carried out with WT and *Bmal1* KO seminiferous tubules that had been fixed in glutaraldehyde. Ultrastructural analyses show a CB with its regular sponge-like structure, and regions that have different electron-density levels, in WT spermatids (1). Conversely, CBs from *Bmal1* KO spermatids display altered shape (2), and fragmentation of their structure (3 and 4). In most cases, a large CB was found adjacent to a small ‘satellite’ fragment. (B) The number of WT, *Bmal1* KO and *Clock* KO round spermatids possessing 1 or 2 or more CBs was determined. Most of the spermatids have one single CB spot. There was no statistical difference among the number of WT, *Bmal1* KO and *Clock* KO round spermatids possessing 1 single CB spots or between the number of *Bmal1* KO and *Clock* KO round spermatids possessing 2 or more CB spots. Scale bars: 1 and 2∶1 µm; 3 and 4∶0.5 µm.

### Electron Microscopy

Testes fragments from *Bmal1* WT and *Bmal1* KO mice were removed and sliced into small pieces, and samples of the germ epithelium were cut and immersed in 2% glutaraldehyde in PBS for 24 hours at 4°C. After fixation, samples were prepared according to standard procedures for osmium tetroxide post-fixation, acetone dehydration, infiltration, embedding, trim, sectioning and uranyl acetate and lead citrate staining. Samples were evaluated using a Philips CM-10 Transmission Electron Microscope and documented using Gatan UltraScan Digital Camera.

**Figure 5:**
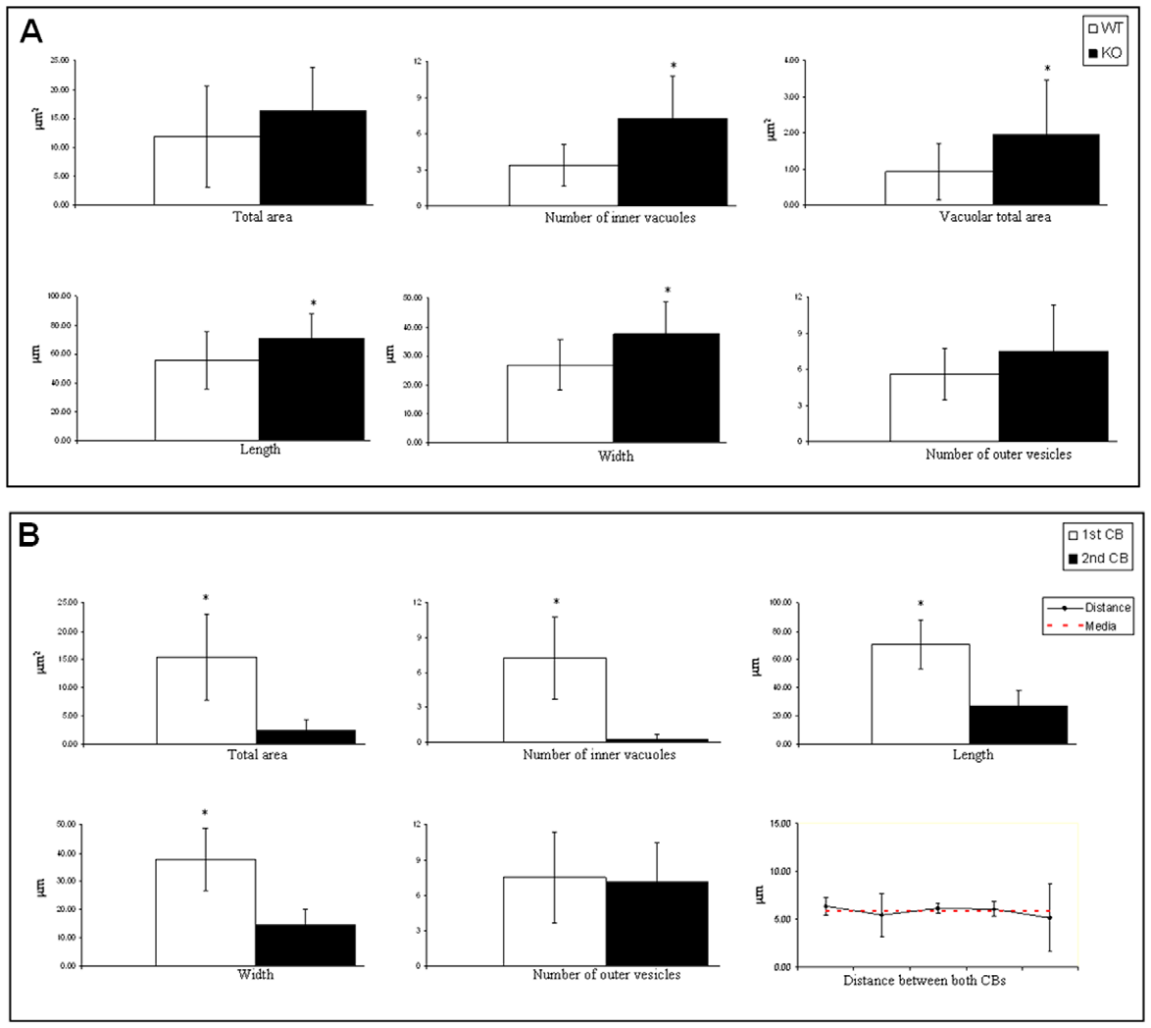
CBs of *Bmal1* KO round spermatids display morphological alterations. (A) Morphological parameters were measured in WT and *Bmal1* KO round spermatids, such as total area, number of inner vacuoles, vacuolar total area, length, width and number of outer vesicles. Data were compared using test t Student and the asterisks (*) indicate statistical differences between WT and *Bmal1* KO CB. Statistical significance was considered with a p≤0.05. (B) Morphological parameters were measured in *Bmal1* KO round spermatids possessing 2 CB spots, such as total area, number of inner vacuoles, vacuolar total area, length, width and number of outer vesicles, and distance between the two CB spots. Data were compared using test t Student and the asterisks (*) indicate statistically significant differences between the large main CB (1^st^) and the ‘satellite’ CB (2^nd^). Statistical significance was considered with a p≤0.05.

**Figure 6:**
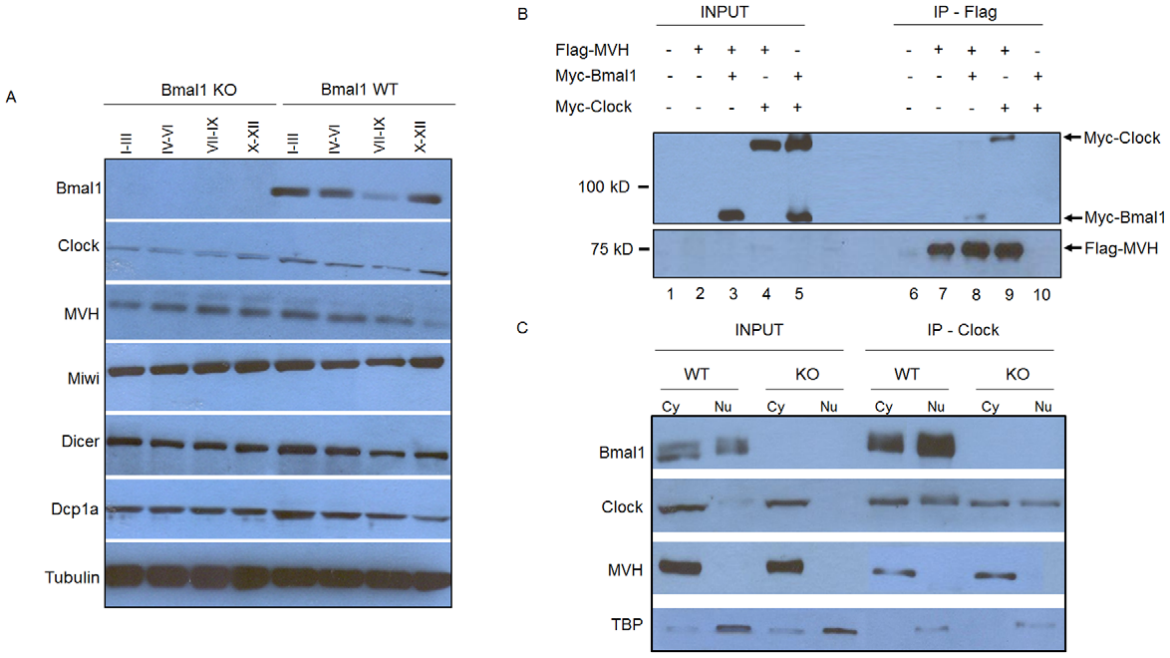
BMAL1 and CLOCK may play an important role during the spermatogenesis process. (A) BMAL1 is differentially expressed during the development of seminiferous tubules. Total proteins from stage-specific seminiferous tubules segments, which were identified by transilluminating dissection under transilumination microscope, were extracted from WT and *Bmal1* KO mice. Extracts were immunoblotted with anti-BMAL1, anti-CLOCK, anti-MVH, anti-MIWI, anti-DICER and anti-DCP1a antibodies. A decrease in BMAL1 levels was detected in stages VII-IX. The amount of CB proteins of *Bmal1* KO specific-stages segments did not follow the same expression pattern as compared to WT mice. Tubulin served as a loading control. (B) Immunopurified (IP) Flag-MVH by M2 Agarose from HEK 293 cells was probed by western blot with the indicated antibodies. Total lysates after co-immunoprecipitation were used as input. (C) In vivo immunoprecipitation (IP) was performed from cytoplasmic and nuclear fractions from seminiferous tubules of *Bmal1* WT and *Bmal1* KO mice with anti-CLOCK antibody. Samples were immunoblotted with the indicated antibodies. Cytoplasmic and nuclear fractions after co-immunoprecipitation were used as input.

### Data Analysis

Two hundred and six round spermatids of *Bmal1* WT mice and 262 round spermatids of *Bmal1* KO mice were analyzed by immunofluorescence and transmission electron microscopy in order to accurately monitor the number of CBs present in round spermatids and determine the percentage of round spermatids possessing one or more CBs. Similarly, 166 round spermatids of *Clock* KO mice were analyzed by immunofluorescence. The percentages of cells with 1 or 2 CBs were compared among the three groups using the chi-square test [25] performed in Statistica 8.0 (Copyright^©^ StatSoft, Inc. 1984–2007), with statistical significance at p≤0.05. Some morphological parameters of CBs were measured in round spermatids of *Bmal1* WT and *Bmal1* KO mice, such as CB’s total area, length and width; number of inner vacuoles, which are low density areas inside of the CBs; vacuolar total area; and number of outer vesicles, which are membrane-bounded structures in the CBs surrounding cytoplasm. In addition, the same morphological parameter plus the distance of both CBs spots in the round spermatids of *Bmal1* KO mice possessing 2 CBs were assessed. All of the mentioned measures were taken from images using Image J-Image Processing and Analysis in Java software, Version 1.40 (http://rsb.info.nih.gov/ij/) for image analysis and each of their mean values were compared for statistical significance (t test) [25]. The t tests were performed in Statistica 8.0 (Copyright^©^ StatSoft, Inc. 1984–2007), with significance level also at p≤0.05.

### Protein Extracts and Immunoblotting

Total proteins were extracted from whole testis or from stage-specific seminiferous tubules segments, which were identified by transilluminating dissection under transilumination microscope [24] from *Bmal1* WT, *Bmal1* KO, *Clock* WT and *Clock* KO mice. Proteins were obtained homogenizing the whole tissues and also the stage-specific seminiferous tubules segments in RIPA buffer (50 mM Tris pH 8.0, 150 mM NaCl, 20 mM EDTA, 15 mM MgCl_2_, 1% NP40) containing protease inhibitors (NaF 5 mM, protease inhibitor cocktail (Roche), and 0.5 mM PMSF). Total proteins were separated by SDS–PAGE and transferred to a nitrocellulose membrane. Membranes were blocked with 5% nonfat milk. Blots were incubated with anti-MVH rabbit polyclonal (1∶8000), anti-MIWI rabbit polyclonal antibody (Cell Signaling – G82) (1∶500), anti-CLOCK goat polyclonal antibody (Santa Cruz Biotechnology, Inc. – S-19) (1∶500), anti-BMAL1 rabbit polyclonal antibody (Abcam – ab93806) (1∶1000), anti-DICER rabbit polyclonal (Santa Cruz – H-212) (1∶500), anti-DCP1a rabbit polyclonal (Abcam – ab66009) (1∶500), and anti-Tubulin mouse monoclonal (Sigma-Aldrich – T5168) (1∶10000) overnight at 4°C. Secondary antibodies used were anti-rabbit IgG-HRP (Cell Signaling –7074) (1∶5000 or 1∶8000), donkey anti-goat IgG-HRP (Santa Cruz – SC-2020) (1∶3000); and rabbit anti-mouse (Millipore – AP160P) (1∶12000). After incubation with secondary antibodies membranes were treated with HRP-enhanced chemiluminescence system (Immobilon Western – Chemiluminescent HRP Substrate – Millipore) to detect proteins.

### *In vitro* and *in vivo* Co-immunoprecipitations Assay

For the in vitro co-immunoprecipitation assay HEK 293 cells (ATCC, Manassas, VA) were cultured in DMEM supplemented with 10% NCS and antibiotics, and transfected with pcDNA3-Flag-MVH, pcDNA3-Myc-CLOCK and/or Myc-BMAL1 plasmids using BioT (Bioland Scientific). Two days after transfection, cells were collected and lysed in modified RIPA buffer (50 mM Tris-HCl pH 8.0, 150 mM NaCl, 5 mM EDTA, 15 mM MgCl2, 1% NP-40, supplemented with protease inhibitor cocktail (Roche), 20 mM NaF, and 5 mM PMSF). Cell lysates were mixed with M2 Agarose (Sigma) overnight at 4°C after centrifugation. The immunoprecipitated products were separated by SDS-PAGE followed by Western blot with the following antibodies after extensive washes: anti-FLAG rabbit polyclonal (1∶10000), anti-MYC mouse monoclonal (1∶5000). For the in vivo co-immunoprecipitation assay, total lysates or cytoplasmic and nuclear lysates from seminiferous tubules of *Bmal1* WT and *Bmal1* KO mice (≈500 µg of proteins per fraction) were incubated with immunoprecipitation (IP) antibody (CLOCK) and incubated overnight at 4°C. Thirty µl of protein G-agarose was then added into each mixture followed by rotating at room temperature for 2 h, centrifuged, washed three times, and resolved on an SDS-PAGE gel followed by Western blot analysis with the following antibodies: anti-CLOCK, anti-BMAL1, anti-MVH, anti-TBP (1∶2500), anti-MIWI (1∶500), anti-EIF4E (1∶5000), and anti-Tubulin (1∶10000).

### Sperm Counting and Motility

Sperm samples from six WT and six *Clock* KO mice were harvested from cauda epididymis following standard procedures [26]. Briefly, cauda epididymis was collected in cold PBS, fat and other tissues were removed, and sperm harvesting was performed in 1 ml non-capacitating MEM media. Sperm concentration (million sperm/ml) and motility was assessed under optical microscope. Cauda sperm motility was subjectively defined from a range of 1 to 3 (1, low motility and 3, high motility) according to the percentage of motile cells.

## Results

### BMAL1 and CLOCK Accumulate in the CB and Ablation of BMAL1 Causes Alterations in the Assembly of the CB

We sought to study the biology of circadian clock proteins in postmeiotic male germ cells. To do so, we performed immunofluorescence on squash preparations to detect the CB in round spermatids from WT, *Bmal1* KO and *Clock* KO mice. Detection of CBs was made using antibodies against two hallmark CB proteins: MVH and MIWI. As expected, the CBs were visualized in the cytoplasm of WT spermatids as single perinuclear spot (Figures 1a and 1d) - green diffuse MVH staining in the cytoplasm is also observed. Strikingly, the CBs were often visualized as two or three perinuclear spots in the cytoplasm of *Bmal1* KO spermatids (Figures 1b and 1e) and *Clock* KO spermatids (Figures 1c and 1f), independently on whether we monitored for MVH or MIWI. This alteration in the number of CB spots prompted us to analyze the subcellular localization of BMAL1 and CLOCK proteins in round spermatids. Immunofluorescence on squash preparations were performed to test if CLOCK and BMAL1 immunostaining may overlap with CB components. Indeed, the CLOCK signal was found to colocalize with MVH in the cytoplasm of WT and *Bmal1* KO round spermatids (Figure 2A), thus confirming the localization of CLOCK in this structure. CLOCK was also detected in the acrosomal region of round spermatids mainly in seminiferous tubules at stages VII-XII of the spermatogenic cycle (data not shown), as previously reported by Alvarez et al. [27]. BMAL1 signal was found to overlap with another CB component, the eukaryotic translation initiation factor 4E (EIF4E) in the cytoplasm of WT round spermatids (Figure 2B), thus confirming the co-localization of BMAL1 with CLOCK. As expected, BMAL1 was not detected in the CB of *Bmal1*-null mice. On the other hand, BMAL1 was also absent in the CB of *Clock*-null animals, a finding that would link BMAL1 to the integrity of the CB. In this respect, we found that the levels of BMAL1 are very low in the testis of *Clock*-null mice (Figure 3B), which could be related to the failure of BMAL1 to localize in the CB of *Clock*-deficient round spermatids. Low levels of BMAL1 were also detected in other tissues of *Clock*-null mice, such as brain and liver [23].

Next we determined how often the lack of *Bmal1* and *Clock* may result in an aberrant number of CB spots in the cytoplasm of round spermatids. We examined CBs from 206 spermatids of WT mice, 262 from *Bmal1*-null mice, and 166 from *Clock*-deficient mice. While 100% of round spermatids from WT mice had a single CB spot, the number of spermatids with a single CB dropped to 87% in the *Bmal1*-null mice and 87.9% in the *Clock*-null mice, while the remaining 13% of the *Bmal1* KO round spermatids and 12.1% of the *Clock* KO round spermatids had two or more CB spots (Figure 4B). No differences were found among the percentages of WT, *Bmal1* KO and *Clock* KO round spermatids having one CB spot (chi-square = 1.15; p = 0.56), or between the percentages of *Bmal1* KO and *Clock* KO round spermatids with two or more CB spots (chi-square = 0.04; p = 0.84). However it was clear that only both of null mice had alterations in the number of CB spots in the cytoplasm of round spermatids.

### Lack of BMAL1 Localization in the CB Produces Morphological Alterations

Since ablation of BMAL1 appeared correlated to the alteration in the number of CB spots, we analyzed the ultrastructure of CBs using transmission electron microscopy (Figure 4A). The CBs of WT round spermatids showed the typical round shape and a sponge-like structure, with regions having different electron-density levels (Fig. 4A1). In striking contrast, the CBs of *Bmal1* KO round spermatids had altered shapes (Fig. 4A2), or were associated with small CB fragments (Fig. 4A3,4). Thus, it appears that the double CB spots observed in *Bmal1* KO round spermatids are result of CB fragmentation rather than duplication of the structure. To determine the extent of the morphological alterations we measured ultrastructure morphological parameters characteristic of the CB, such as: total area, number of inner vacuoles, vacuolar total area, length, width and number of outer vesicles. We found higher values for all parameters in the CBs of *Bmal1* KO round spermatids, suggesting that lack of BMAL1 will induce an increase in the accumulation of material in the CB (Figure 5A). We also measured total area, number of inner vacuoles, length, width, number of outer vesicles and distance of CBs spots of *Bmal1* KO round spermatids displaying CB fragmentation. We found statistical differences in all parameters analyzed, except for the number of outer vesicles, which may indicate that despite of the difference in size, the smaller CB fragment could be still active (Figure 5B). The two CB fragments were always found to be closely associated, with a mean distance of 5.83 µm and a small range of variation (Figure 5B).

The abundance of specific proteins known to be essential CB components was examined in whole testis lysates of *Bmal1* KO, *Clock* KO mice and their respective WT littermates. No differences were found in the amount of MVH, MIWI, DICER and DCP1a (Figure 3A). This finding supports the notion that the morphological alterations caused by BMAL1 ablation are due to a reorganization of the CB rather than to a duplication of this structure, which would presumably require an increase in the overall levels of CB components.

### BMAL1 and CLOCK may Play a Role in the Assembly and Physiology of the CB

We examined the developmental expression of BMAL1 and CLOCK in parallel with some essential CB components, such as MVH, MIWI, DICER and DCP1a in both WT and *Bmal1* KO testis (Figure 6A). We observed a notable decrease in the expression of BMAL1 in seminiferous tubules at stages VII-IX of the spermatogenic cycle, while CLOCK expression was mostly constant in both WT and mutant mice. An expected, slight variation in the expression of essential proteins that constitute the CB was observed along the spermatogenic cycle stages in WT testis. This variation was not observed in the same spermatogenic cycle of *Bmal1* KO testis. This result prompted us to investigate whether CLOCK and BMAL1 could physically interact with MVH. To do so, we transiently expressed and immune-purified Flag-MVH, Myc-BMAL1 and/or Myc-CLOCK in HEK 293 cultured cells. We found that both Myc-BMAL1 and Myc-CLOCK co-precipitated with Flag-MVH when co-expressed (Figure 6B, lanes 8 and 9). The interaction with MVH was also tested in vivo. We performed co-immunoprecipitation assays using an anti-CLOCK antibody in cytoplasmic and nuclear fractions from seminiferous tubules of *Bmal1* WT and *Bmal1* KO mice. MVH co-immunoprecipitated with CLOCK in both *Bmal1* WT and *Bmal1* KO cytoplasmic fractions (Figure 6C) indicating that the presence of BMAL1 is not required for the interaction between CLOCK and MVH in vivo. We also performed a control co-immunoprecipitation assay to establish whether CLOCK interacts with others CB’s components in seminiferous tubules of *Bmal1* WT and KO mice, such as MIWI and EIF4E. We could observe that CLOCK also interacts with those CB’s components, and the results are shown in the Figure S1.

## Discussion

The role of the circadian clock and of clock proteins has been studied extensively in both central and peripheral tissues [18]. Yet, little is known about the role of circadian clock proteins in the spermatogenesis process in mammals. Distribution of CLOCK and BMAL1, the central clock proteins, was already described in the male germ epithelium. BMAL1 localizes mainly in Leydig cells but it can also be found in the nucleus and cytoplasm of germ cells [20]. CLOCK was observed having a higher expression level in the cytoplasm of round spermatids [27]. Our results show that there is a significant decrease in BMAL1 expression levels in seminiferous tubules at stages VII-IX of the spermatogenic cycle, which surprisingly coincides with the timing of increase in the expression of PER1, a protein whose activity is controlled by CLOCK:BMAL1 complex [18]. We have also shown that CLOCK and BMAL1 colocalize in the CB and the absence of BMAL1 in this cytoplasm germ cell structure leads to its morphological alterations. This finding raises questions as to whether components of the circadian clock play an integral part in testis function.

The CB is considered a highly specialized structure that acts as a subcellular coordinator of various RNA-processing pathways, and centralizes post-transcriptional mRNA control in the cytoplasm of haploid male germ cells [28]. This notion is based on the evidence that it contains factors mediating mRNA degradation and translational repression such as miRNAs, piRNAs, the XRN1 RNase, decapping enzymes, argonaute proteins, RNA helicases, and the Y-box protein MSY2 [8], [29]. It has also been proposed that the CB functions as a degradation site based on some of its aggresomal features [12]. Another view implicates the CB as a central site in degradation pathways where unnecessary DNA, RNA and proteins are digested [30]. In addition, by the end of the spermiogenesis process, the CB moves slowly through the cytoplasm to the caudal pole of the nucleus reaching the base of flagellum, where it is enclosed compactly by small vesicles and its matrix becomes denser. Gradually the CB decreases in size and finally disappears [31]. In our study we found that ablation of BMAL1 leads to morphological alterations in CB’s structure, generating fragmentation of its structure. Thus, BMAL1 appears to function as an integral component of the CB, a view that is supported by its differential expression in seminiferous tubules at different spermatogenic cycles and its physical association with MVH, an important CB component. We speculate that BMAL1 and CLOCK localize in the CB to participate in CB assembly and physiology, rather than being targeted for degradation.

Higher levels of BMAL1 expression in seminiferous tubules at stages I–VI followed by a decrease in this expression in seminiferous tubules at stages VII–IX (Figure 6A), in addition to the detection of CLOCK and BMAL1 in the CB of post-meiotic round spermatids (Figures 2A and B), could be predictive of a pre-meiotic role for CLOCK and BMAL1. Absence of BMAL1 in testicular spermatogenic cells of *Bmal1* KO or decreased expression in *Clock* KO mice (Figures 2A, B, and 3B) may cause an unbalance in the pre-meiotic silencing of mRNAs targeted to be translated in the last steps of the male germ cell differentiation. This unbalance would somehow increase the volume of the material accumulated in the CB causing all the morphological alterations observed in CBs of *Bmal1 KO* mice in this study. We observed no alterations in the expression levels of the CB’s main proteins neither in the seminiferous tubules of *Bmal1* KO nor *Clock KO* mice, such as MVH, MIWI, DCP1a and Dicer (Figure 3A), which could be indicating that accumulation of those proteins are not the cause of the increased CB size in *Bmal1* KO mice.

Our results suggest that BMAL1 ablation may cause an imbalance in the organization of the CB machinery involved in mRNA silencing and translational regulation, leading to the an increased size of the CB and also fragmentation of its structure (Figure 4A), as mentioned before. It is tempting to speculate if this defect in CB physiology could be related to the fertility defects observed in *Bmal1* KO mice [20], [19]. It has been shown that infertility or reduced fertility problems showed by *Bmal1* KO and *Clock* KO mice, respectively, are not driven by direct defects in the spermatogenesis [20], [19]. Even then, the small percentage of reduction in the sperm counting and motility found in the *Bmal1* KO [20] and *Clock* KO (Table S1) mice can be correlated with the small percentage of round spermatids showing morphological alterations in the CB assembly (Figures 4B, 5A and B). Fully active CBs are essential to the differentiation of round spermatids to mature spermatozoon. Although endocrine or systemic dysregulation in *Clock* and *Bmal1* KO animals could be partially involved in the CB morphologic and physiologic impairment shown here, we provide clear evidence that these two circadian proteins are localized in the CB and physically interacting with CB components suggesting a potential functional role in the CB physiology. Interestingly, ablation of the *miwi* gene in the mouse also leads to fragmentation of the CB, and spermatogenesis defects [28]. MIWI is a critical component of the CB, playing a critical role in piRNAs metabolism, and also interacts with MVH. An additional and noteworthy observation relates the role of CLOCK to the CB morphology and physiology. Our results in *Clock*-null mice indicate that CLOCK is essential in controlling the levels of BMAL1 expression and its location within the CB (Figure 2B and 3B).

The presence of BMAL1 and CLOCK as molecular components of the CB and their interaction with MVH, a germ cell-specific DEAD-box RNA helicase, as well as with other CB components (such as MIWI and EIF4E) could also be related to the post-meiotic transcriptional/translational reactivation role that the CB plays in round spermatids. Interestingly, a recent report indicates that CLOCK and BMAL1 play a role in transcriptional activation after viral infection [32]. BMAL1 and CLOCK were found to be located within ND10 bodies, a complex of proteins where viral DNA is thought to be silenced. CLOCK and BMAL1 were shown to interact with the ICP0 protein, a multifunctional transcriptional regulator, which interacts with the UbcH5a-conjugating enzyme to degrade PML and SP100, two key components of ND10 bodies. Additionally, this protein complex also plays a role in viral chromatin remodeling to enable effective transcription [32].

Specific molecular components of the CB, such as MIWI and piRNAs, have been recently linked to mitotic promotion of chromosome condensation and segregation by facilitating robust chromosomal localization of condensin I in the *Drosophila* germ line [33]. Furthermore, the CB has been linked to synapsis and XY body formation during meiosis by interacting with the dense body, a male-specific organelle associated with meiotic processes [8]. These findings suggest that CB molecular components may play previously unappreciated functions during mitotic and meiotic cell division. It is tempting to speculate that these may include meiotic silencing and/or epigenetic modifications, since small RNA pathways are involved in similar mechanisms in other systems [34], [35]. Besides the previously stated potential pre-meiotic mRNA silencing unbalance in the absence of BMAL1, another possible function of the CB components could be their involvement in the crossing-over process during meiosis since a DNA helicase involved in DNA repair and recombination is a component of a piRNA complex in rat testis [36]. The presence of CLOCK and BMAL1 as molecular components of CBs could also be related to these above mentioned functions. Indeed, CLOCK has been shown to interact with the RNA processing protein INTS7 and the recently characterized protein RHINO to sites of DNA damage, which suggests that this complex has a more direct role in the DNA damage response (DDR) [37]. Our findings represent a critical first step towards the understanding of the physiological roles that the circadian proteins BMAL1 and CLOCK have in reproduction and fertility.

## Supporting Information

Figure S1 ***In vivo*** **co-immunprecipitation (Co-IP) was performed from 750 ug of total protein from seminiferous tubules of WT and** ***Bmal1*** **KO mice with anti-CLOCK antibody.** Samples were immunoblotted with BMAL1 antibody (abcam - ab93806) (1/2500), CLOCK antibody (Santa Cruz Biotechnology, Inc. - SC6927) (1/500), tubuline (Sigma - T5168) (1/10000) and chromatoid body related proteins such as MVH antibody (1/8000), MIWI antibody (Cell signaling - G82-2079) (1/500) and eIF4E (Abgent - AM1852a) (1/5000).
(TIF)

Table S1 **Sperm counting and motility of** ***Clock*** **WT and KO mice.**
(DOCX)
