# Supplementary figures and images for: Circadian Proteins CLOCK and BMAL1 in the Chromatoid Body, a RNA Processing Granule of Male Germ Cells
Source: PLoS One. 2012 Aug 10;7(8):e42695. doi: 10.1371/journal.pone.0042695 (PMC3416844; doi:10.1371/journal.pone.0042695)

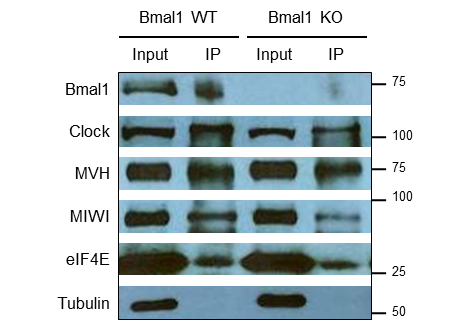

Supplement: Figure S1 — In vivo co-immunprecipitation (Co-IP) was performed from 750 ug of total protein from seminiferous tubules of WT and Bmal1 KO mice with anti-CLOCK antibody. Samples were immunoblotted with BMAL1 antibody (abcam - ab93806) (1/2500), CLOCK antibody (Santa Cruz Biotechnology, Inc. - SC6927) (1/500), tubuline (Sigma - T5168) (1/10000) and chromatoid body related proteins such as MVH antibody (1/8000), MIWI antibody (Cell signaling - G82-2079) (1/500) and eIF4E (Abgent - AM1852a) (1/5000). (TIF) [file pone.0042695.s001.tif]
