# Supplementary material for: Circadian Proteins CLOCK and BMAL1 in the Chromatoid Body, a RNA Processing Granule of Male Germ Cells
Source: PLoS One. 2012 Aug 10;7(8):e42695. doi: 10.1371/journal.pone.0042695 (PMC3416844; doi:10.1371/journal.pone.0042695)
Supplement: Table S1 — Sperm counting and motility of Clock WT and KO mice. (DOCX) [file pone.0042695.s002.docx]

Table S1

| Genotype | Cauda conc. (million sperm/ml) | Cauda sperm motility (range 1-3) |
| --- | --- | --- |
| *Clock* WT | 22.64 | 2.17 |
| *Clock* KO | 17.03 | 1.83 |
|  |  |  |
| T-test | 0.57 | 0.57 |
